# Obesity in childhood: associations with parental neglect, nutritional habits, and obesity awareness

**DOI:** 10.3389/fnut.2024.1430418

**Published:** 2024-07-02

**Authors:** Tuba Onay, Utku Beyazıt, Aslı Uçar, Aynur Bütün Ayhan

**Affiliations:** ^1^Department of Nutrition and Dietetics, Faculty of Health Sciences, Bandirma Onyedi Eylul University, Balıkesir, Türkiye; ^2^Child Development Department, Kumluca Health Sciences Faculty, Akdeniz University, Antalya, Türkiye; ^3^Department of Nutrition and Dietetics, Faculty of Health Sciences, Ankara University, Ankara, Türkiye; ^4^Child Development Department, Faculty of Health Sciences, Ankara University, Ankara, Türkiye

**Keywords:** obesity, obesity awareness, parental neglect, nutritional habits, nutrition education

## Abstract

**Background:**

The relationships underlying the dynamic between obesity and parental neglect in terms of nutritional habits and obesity awareness are unclear. Parental neglect remains a significant subject of concern that needs to be examined in the context of obesity.

**Methods:**

The aim was to examine the relationships between childhood obesity, parental neglect, children’s eating habits and obesity. The study group consisted of 404 children and their parents from Ankara, Turkiye. As data collection tools, an Individual Information Form, Obesity Awareness Scale, the Parents Form of the Multidimensional Neglectful Behaviors Scale were administered. In addition, information on the children’s body mass indexes was obtained by anthropometric measurements and the findings were recorded on the questionnaires of each child.

**Results:**

It was found that 98 (24.3%) of the children included in the study were overweight and 63 (15.6%) were obese. The results of the multinomial logistic regression analysis indicated that in the underweight and overweight group, the parents’ perception of their child’s weight predicted body mass index in children, and in the obese group, along with the parents’ perception of their child’s weight, the age and gender of the child, eating fast, obesity in the family and parental neglect were also predictors.

**Conclusion:**

Practitioners such as nurses, dietitians and child developmentalists working in schools should consider weight problems in children as one of the indicators of parental neglect and should implement interventive efforts to enhance parental supervision of children at risk.

## 1 Introduction

Childhood obesity is considered to be one of the most important public health problems in modern society. The World Health Organization (1) has reported that more than 340 million children between the age of 5 and 19 are overweight or obese and the obesity prevalence in children is expected to double worldwide by 2030 (2). According to the Turkey Childhood Obesity Survey, one in 10 children is obese (3). Environmental factors play a role in this wide prevalence of obesity in addition to genetic and hormonal etiology (4, 5). The obesogenic environment encourages excessive food intake and reduces physical activity, which leads to the spread of the obesity epidemic (6, 7). As the basis for the acquisition of eating habits, the family constitutes one of the main factors related to the obesogenic environment (8). Dominant eating preferences and nutritional culture in this environment are among the physical environmental factors that have an effective role in the formation of obesity (9). In addition, the family is generally the primary environment in which the child’s basic psychological and social needs are met. Therefore, as an emotional environment, the family also affects the child’s habits (10, 11).

In this primary environment, those responsible for meeting the basic needs of the child are primarily the parents. Their failure or insufficiency to adequately meet the physical and socio-emotional needs of children is defined as neglect, which affects the development of the child in a wide variety of ways (12). One of the ways neglect occurs physically is via insufficient or excessive nutrition (13). In this type of neglect, parents either do not meet the nutritional needs of their children adequately or at all or force the child to eat more by ignoring their needs (14). Neglect may sometimes occur in the form of a lack of supervision about nutritional habits. In other words, parents do not set rules and establish control that will ensure the child’s healthy nourishment (15). In other words, the parents’ inability to set limits on the child’s nutrition, insufficient control of the child’s eating habits, lack of interest in where, how and what the child eats, allowing the child to consume fast food continuously and not teaching the child the right and wrong behaviors about nutrition can be considered as neglect (16, 17). Studies have shown that the risk of overeating and obesity in children particularly increases with insufficient parental supervision (18) and coercive controlling practices such as insistence on eating (19). Parents’ attitudes and behaviors towards their children’s nutiriton are also shaped by their perceptions of body mass, which affect their attitudes and behaviors towards nutritional habits (20). Parents’ critical comments about children’s body, weight and physical appearance in the family environment, perceiving their weight to be more or less than it is, encouraging them to eat more or restricting their eating may affect children’s perceptions of their own body and weight. In other words, parents’ perceptions and attitudes may affect children’s awareness of their own body weight and eating habits (21, 22) and this may cause children to perceive themselves to have a healthier weight than they actually do, thus making weight control difficult and negatively affecting their quality of life (23). In a study conducted by Yüksel and Akıl (24), it was shown that a relationship exists between obesity awareness and sedentary lifestyle, particularly in adolescence; in other words, adolescents with high obesity awareness have higher physical activity levels and less uncontrolled eating behaviors. Therefore, in line with these arguments, it can be suggested that obesity awareness can positively affect the nutritional behaviors of children.

On the other hand, neglect sometimes arises with attitudes and behaviors such as ignoring the child emotionally, and not providing the children with the love and affection they need (25). Children who experience emotional rejection may regard themselves as being responsible for this situation, and consequently experience feelings of guilt, emotional deprivation and psychological regression (26, 27). A number of studies in the literature have provided evidence that emotional deprivation affects children’s eating behavior, suppresses their appetite and causes extremely low weight, or leads to overeating by causing emotional hunger (28–30). In two separate meta-analysis studies, it was indicated that various eating disorders are seen in individuals exposed to childhood neglect (31), and that 53.3% of adults with an eating disorder suffered from emotional neglect in their childhood (32). Besides eating disorders, parental neglect may also be the cause of other psychological problems such as depression and anxiety (33). Hence, it can be argued that neglect may have a direct effect on the nutrition and weight of children, as well as indirectly affecting the body mass of children by causing psychological difficulties that may affect appetite and physical activity levels. The findings that emotional problems caused by parental neglect can affect metabolism and lead to neuro-endocrine responses that change physical activity and appetite can be regarded as supportive of this argument (34).

As a result, when the literature on obesity is examined in general, it is seen that a wide variety of studies have been conducted on the risk factors of obesity. However, these studies have generally focused on the physiological aspects of obesity and have not focused on the psycho-social factors such as family and individual awareness. We know that obesity is a multifactorial disease. However, there are limited studies on the dimensions that affect obesity-related mood changes and mood changes that lead to obesity. In this context, the question of what is the impact of the family on the emotional state of the child comes to mind. Considering that parental attitudes can cause eating disorders; the effect of parental neglect on obesity is also a matter of curiosity. In particular, the relationships underlying the dynamic between obesity and parental neglect in terms of nutritional habits and obesity awareness are unclear. While child neglect is mostly associated with malnutrition and underweight body mass, the possible relationship between attitudes and behaviors of parents that may lead to obesity, children’s eating habits, and parental neglect remains a significant subject of concern that needs to be examined. It is thought that the assessment of these relationships may contribute to a better and deeper understanding of the underlying causes of obesity. From this point of view, in the present study, the purpose was to examine the relationships between childhood obesity, parental neglect, children’s eating habits and obesity awareness. Study; Because Ankara is the capital of Turkey, researchers are in Ankara, it is among the provinces with the highest number of schools and population in Turkey, Ankara is in a central place due to its location, and many people live in Ankara in terms of socio- demography. It was held in Ankara because it has a high potential to represent Turkey.

## 2 Material and method

### 2.1 Participants

The study which is planned as a cross-sectional descriptive research consisted of 404 children and their parents who were attending eight separate secondary and nine separate high schools affiliated to the Directorate of National Education in the city center of Ankara. In order to determine the schools to be included in the study, information was obtained from the Ministry of National Education about the schools in the city center. In line with the information obtained, schools with population structures and socio-economic characteristics that were thought to represent the population culturally and economically were selected by using stratified simple random sampling (35). The study included families and their children who had consented to participate and signed the consent form. The study included families and their children who spoke Turkish fluently, signed the voluntary consent form, and had children between the ages of 10 and 15. Those who did not meet the inclusion criteria were excluded from the study. Those who fell outside the inclusion criteria were not accepted into the study. The socio-demographic characteristics of both the children and their parents are presented in Table 1.

**Table 1:** Socio-demographic characteristics of the children and their parents.

	The socio-demographic characteristics	*n* %	
Children	Gender		
	Female	242	59.9
	Male	162	40.1
Parents	Gender		
	Female	298	73.8
	Male	106	26.2
	*Educational Status*		
	Graduate of primary school or less	44	10.9
	Graduate of secondary school	55	13.6
	Graduate of high school	138	34.2
	University graduate	167	41.2
	*Income Level*		
	Low	67	17.1
	Average	249	61.6
	High	76	18.8

Missing values are excluded in the analyses.

When the children’s socio-demographic characteristics are examined, it is seen that they were aged between 10 and 16 with an average of 12.51 ± 15.53. A total of 242 (59.9%) of them were girls and 162 (40.1%) were boys. With regard to the parents, 298 (73.8%) of them were mothers and 106 (26.2%) were fathers. Their ages varied between 25 and 65 with an average of 41.62 ± 58.85. In terms of the parents’ educational status, 44 (10.9%) of the parents were graduates of primary school or had not graduated from any school, 55 (13.6%) were graduates of secondary school, 138 (34.2%) were graduates of high school, and 167 (41.3%) were graduates of university. In terms of the income level, 67 (17.1%) of the parents had a low income, 249 (61.6%) of them had an average income and 76 (18.8%) had a high income.

### 2.2 Instruments

In the study, the “Individual Information Form” was implemented to determine the socio-demographic information and eating habits of the children and their parents. The Obesity Awareness Scale was administered to the children in order to assess their awareness of obesity, and the Parents Form of Multidimensional Neglectful Behaviors Scale was implemented to the parents to determine their level of neglect towards their children. In addition, information on the children’s body mass indexes was obtained by anthropometric measurements and the findings were recorded on the questionnaires of each child.

Individual Information Form. Separate information forms for the children and their parents were prepared by the researchers. Both forms consisted of two parts, the first of which was prepared for children and included questions about their age and gender, while the second part included questions about their nutritional habits such as whether they regularly eat breakfast, lunch and dinner every day, their speed of eating, whether they eat junk food and if so at which frequency, whether their parents insist that they eat and whether they do exercise/sports. On the other hand, the form for parents included socio-demographic questions about age, gender, income and education level, in addition to questions about whether there was an overweight or obese person in the family, the perceptions of the parents about the weight of their child, whether the parents immediately bought fast-food products the child saw on television advertisements and the frequency of the child’s eating in fast-food restaurants.

Obesity Awareness Scale. The scale was developed by Allen (25) in order to evaluate the awareness levels of children and adolescents about obesity and the risk factors of obesity such as nutrition and physical activity. The scale consists of 20 4-point Likert-type items ranging from strongly disagree (1) to completely agree (4) as well as 3 subscales, which are obesity awareness (i.e., “Childhood obesity is increasing in my school, in my community and in my country”), nutrition (i.e., “Overeating at every meal is a major cause of obesity”) and physical activity (i.e., “It is important to do at least 60 min of exercises every day to be healthy”). The scale was adapted into Turkish by Kafkas and Özen (36). In the validity and reliability analyses of the Turkish form, the coefficient of internal consistency for the whole scale was calculated as 0.83. For the present study, the coefficient of internal consistency was determined to be 0.79.

Multidimensional Neglectful Behaviors Scale Parents Form. The scale was developed by Kaufman et al. (37) in order to assess the neglectful behaviors of parents towards their children. The original form of the scale consists of 45 4-point Likert-type items with responses ranging from almost never (1) to always (4). The scale was adapted into Turkish by Beyazıt and Ayhan (38). In the reliability and validity study of the Turkish form, the scale was reduced to a total of 21 items in five sub-dimensions, which are physical (i.e., “I take my child to the doctor for health checks.”), supervisory (i.e., “I know the friends my child is with.”) and cognitive (i.e., “I read a book with my child.”) neglect and abandonment (i.e., “I leave my child alone for a long time in environments such as stores, markets, etc.”) and insufficient protection (i.e., “I fight with my wife, throw things in environments where my child can see.”). The coefficient of internal consistency of the Turkish version of the scale was determined as 0.68. Scores that can be obtained from the entire scale range from 0 to 69 and high scores indicate high levels of parental neglect. For the present study, the coefficient of internal consistency was determined to be 0.92.

Body Mass Index (BMI). In the study, the body mass index of children was calculated with their body weight and height. Anthropometric measurements were taken on students safely and precisely in a separate room outside the classroom environment. In this context, BMI was calculated by determining the ratio of the measured weight (kg) to the square of the height (m). Afterwards, BMI categories were identified by the z-score determined by the age and gender of the children. For this purpose, the classification system (<-1SD: thin; ≥-1SD- ≤ +1SD: normal; > + 1SD- ≤ +2SD: overweight and > +2SD: obese) proposed by the World Health Organization (38) was adopted. The body weights of the students were measured with a precision-adjusted Digital Glass Scale after removing any thick clothes and shoes. Measurements were made by placing the scale on a flat, horizontal and hard floor. The students’ height was measured with a stadiometer while their feet were side by side and their head was in the Frankfort plane. Measurements were recorded in cm and with an accuracy of 0.1 cm (39). Accessories and shoes were removed when measuring the height.

### 2.3 Procedure

In the study, necessary permissions were initially obtained from the authors of the data collections instruments which were planned to be administered. Afterwards, ethics committee approval and institutional permissions to implement the study at schools were also acquired. After all the required permissions were obtained, the implementation process was planned with the school administrators. In the implementation procedure, the classroom teachers were informed about the aim and content of the research. In addition to this, the questionnaire sent to the parents, the study also includes an informed consent form containing explanatory information about the study for both parents and student also. In the forms, the content and purpose of the study were explained to the parents and their consent to participate in the study with their children was requested. One week later, the schools were revisited and the forms and the questionnaires sent by the families were collected. During the implementation phase, 737 forms were distributed to be delivered to the parents, 420 of which were ultimately completed and returned. Forms were administered to children who agreed to take part in the study, and then anthropometric measurements were made in a separate room in the school In the evaluation of the data, the forms belonging to 16 children were excluded from the study due to the high number of omitted items, and as a result, the data of 404 children were included in the analyses.

Before commencing the analysis, the collected data were examined in terms of the assumption of normality hypothesis. For this purpose, skewness and kurtosis values were examined. As a result, it was determined that the values were in the range of ±2 and it was concluded that the distribution of the data was normal. Therefore, the Pearson test was conducted in the correlation analysis and the ANOVA test was performed in the comparison of the variables. In terms of the prediction of the children’s body mass index categories by eating habits, obesity awareness and parental neglect, a multinomial logistic regression analysis was conducted. The Omnibus test was conducted regarding the significance of the regression model applied. In addition, in order to examine the fitness of the data to the regression model, the standard error coefficients were examined. and as a result, it was found that all standard values were below 2. As it is accepted that coefficient values below ±2 indicate that the sample means accurately represent the population’s means, it was concluded that the data fit to the model and the predictions were accurate (40). Study analyses were performed by using the SPSS 20 statistical package program.

## 3 Results

The minimum and maximum scores, arithmetic means and standard deviations of the measures implemented in the study are shown in Table 2. As shown in Table 2, the average Body Mass Index Score of the children is 21.22 ± 3.98 kg/m2. The scores vary between 13.34 and 37.24 kg/m2. In terms of the Obesity Awareness Scale, the average score is 55.35 ± 8.67, and the scores vary between 20 and 80. The average score of the Multidimensional Neglectful Behavior Scale is 32.64 ± 6.23. The scores of the children vary between 18 and 56.

**Table 2:** Arithmetic means and standard deviations of children’s body mass index, obesity awareness scale and multidimensional neglectful behavior scale.

Scores	*n*	Minimum	Maximum	x̄	SD
Body mass index	404	13.34	37.24	21.11	3.98
Under weight	25	13.34	18.14	15.42	1.24
Normal weight	218	15.23	23.78	18.93	1.74
Overweight	98	19.71	27.62	23.3	2.8
Obese	63	22.38	37.24	27.43	2.8
Obesity awareness scale	404	20	80	55.35	8.67
Multidimensional neglectful behavior scale	404	18	56	32.64	6.23

The results related to the comparison of the Children’s Obesity Awareness Scale and Multidimensional Neglectful Behavior Scale scores according to their Body Mass Index are shown in Table 3. As indicted in Table 3, the Body Mass Index scores differ significantly according to the scores of the Obesity Awareness Scale [F(3–400) = 2.879, *p* < 0.05]. Further analysis involving the Tukey test revealed that the obesity awareness scores of children who are overweight (56.42) are higher than those who are obese (52.74). On the other hand, the Body Mass Index scores of children do not differ significantly according to the scores of the Multidimensional Neglectful Behavior Scale [F(3–400) = 1,841, *p* > 0.05].

**Table 3:** The comparison of the obesity awareness scale and multidimensional neglectful behavior scale scores of children according to their body mass index.

Measures	Variance	Sum of squares	df	Mean square	F	*p*	Significant differences
Obesity awareness scale	Between groups	640.387	3	213.462	2.879	0.036^*^	Overweight-obese
	Within groups	29659.228	400	74.148			
	Total	30299.616	403				
Multidimensional neglectful behavior scale	Between groups	213.427	3	71.142	1.841	0.139	–
	Within groups	15453.531	400	38.634			
	Total	15666.958	403				

^*^, *p* < 0.05.

The correlations among the children’s Body Mass Index, the scores of the Obesity Awareness Scale and the Multidimensional Neglectful Behaviors Scale are presented in Table 4. As shown in Table 4, the children’s Body Mass Index scores are negatively correlated with the scores of the Obesity Awareness Scale’s Nutrition subscale (*r* = −0.120, *p* < 0.05). The Multidimensional Neglectful Behavior Scale scores are negatively correlated with the scores of the Obesity Awareness (*r* = −0.120, *p* < 0.05), Nutrition (*r* = −0.145, *p* < 0.05), Physical Activity (*r* = −0.237, *p* < 0.01) subscales and the total scores of the Obesity Awareness Scale (*r* = −0.192, *p* < 0.01). On the other hand, the total scores of the Obesity Awareness Scale are positively correlated with the scores of the Obesity Awareness (*r* = 0.856, *p* < 0.01), Nutrition (*r* = 0.832, *p* < 0.01), and Physical Activity (*r* = 0.822, *p* < 0.01) subscales.

**Table 4:** The Pearson correlation coefficients of the body mass index, obesity awareness scale and multidimensional neglectful behaviors scale.

Total scale scores	1	2	3	4	5	6
Body mass index	–					
Obesity awareness subscale	−0.010	–	–	–	–	
Nutrition subscale	−0.120^*^	0.525^**^	–			
Physical activity subscale	−0.079	0.542^**^	0.597^**^	–		
Obesity awareness scale	–0.077	0.856^**^	0.832^**^	822	–	
Multidimensional neglectful behavior scale	0.099	–0.120^*^	–0.145^**^	–0.237^**^	–0.192^**^	–

^**^, *p* < 0.01; ^*^, *p* < 0.05.

The multinomial logistic regression analysis results for the prediction of the children’s body mass index categories by eating habits, obesity awareness and parental neglect are shown in Table 5. According to the results of the Omnibus test in the multinomial logistic regression analysis shown in Table 5, the logistic regression model with the independent variables indicates significance (−2 Log Likelihood = 669.671, x^2^ = 250.200, df = 75, *p* < 0.001). According to the Cox-Snell test, the independent variable explains 46% of the variation in the dependent variable, while the independent variable explains 51% of the variation according to Nagelkerke test. As a result of the analysis, it was determined that standard error coefficients of the significant variables in the variable set were below 2. As a result of the Wald statistics, it was observed that children’s body mass was predicted by parents’ perception of the child’s weight (Wald = 11.733, *p* < 0.001) in the underweight versus normal body mass category. In the overweight category, body mass was also predicted by the parents’ perception of the child’s weight as underweight (Wald = 6.061, *p* < 0.05) and overweight (Wald = 14.673, *p* < 0.05). On the other hand, in the obese category, age (Wald = 4,236, *p* < 0.05) and gender (Wald = 8,418, *p* < 0.001) of the child, earing fast (Wald = 4,507, *p* < 0.05), the presence of obesity in the family (Wald = 4,492, *p* < 0.05), parents’ perception of the child’s weight as overweight (Wald = 46.178, *p* < 0.001) and neglectful behaviors (Wald = 4,700, *p* < 0.03) predicted body mass of children.

**Table 5:** Results of multinomial logistic regression analysis related to the prediction of children’s body mass index categories by eating habits, obesity awareness and parental neglect.

Body mass categories	Variables	B	St. error	Wald	*p*	95% Confidence interval	
						Lower	Upper
Underweight	Parents’ perception of child’s weight (underweight)	2.059	0.601	11.733	0.001	2.413	25.479
Overweight	Parents’ perception of child’s weight (underweight)	−1.892	0.769	6.061	0.014	0.033	0.680
	Parents’ perception of child’s weight (overweight)	2.107	0.550	14.673	<0.001	2.797	24.152
Obese	Age	−0.310	0.151	4.236	0.040	0.546	0.985
	Gender (girls)	−1.238	0.437	8.418	0.004	0.120	0.663
	Eating fast	1.036	0.488	4.507	0.034	2.818	1.083
	Obesity in the family	1.054	0.497	4.492	0.034	1.083	7.609
	Parents’ perception of child’s weight (overweight)	4.030	0.593	46.178	<0.001	17.600	179.961
	Parental neglect	0.075	0.035	4.700	0.030	1.007	1.155

Only the significant *β* values of each model are presented. Reference variable, Normal body mass.

## 4 Discussion

In the present study, the aim was to examine the relationships between childhood obesity, parental neglect, children’s eating habits and obesity awareness. The findings indicated that, of the 404 children included in the study, 98 (24.3%) were overweight and 63 (15.6%) were obese. According to the Evaluation of Nutritional Status and Habits Research of the Turkish Ministry of Health (41) the obesity rate among children and adolescents aged 6–18 in Turkey is 8.2%. When the Turkey Health Survey published by the Turkish Statistical Institute (42) is examined it is seen that the rate of overweight school-age children increased to 21.1% in 2019. The finding in the present study is in line with this upward trend.

Obesity; it is a multifactorial disease affected by many factors such as genetics, environmental factors, alcohol, smoking, physical activity and psychology (43). In this study examining the relationship between childhood obesity and parental neglect, when children’s obesity awareness and parental neglect were compared according to children’s body mass index, it was seen that obese children’s obesity awareness was slightly less than other children, and this difference was significant between overweight and obese children. Obesity awareness refers to the perceptions, attitudes, and experiences of obese individuals about their own body, and especially the awareness of their current weight status. Studies on obese adults have revealed that many individuals do not perceive themselves as overweight or obese and consider themselves to have a healthier weight than they do (44, 45). On the other hand, in a study by Liu et al. (46), it was determined that the self-awareness of obese children regarding their weight was lower than that of children with normal weight. A lack of awareness of obesity or not accepting being overweight/obese hinders healthy lifestyle changes. Accurate perception of body weight enables awareness and acceptance of current weight status and is important for body weight control and prevention of weight-related diseases (47).

In the present study, it was found that the body mass index of children was negatively correlated with their nutrition awareness. Although this result was significant, the correlation was weak. When the relationship between body mass index and obesity awareness is examined, the findings in the literature are mixed. In a study conducted by Alasmari et al. (48), which included 528 children, no significant relationship was found between body mass index and obesity awareness. On the other hand, in the study of Yıldırım et al. (49), which included 300 adolescents, it was found that the nutritional awareness level of adolescents with low body mass was low, whereas the level of nutritional awareness of adolescents with a normal body mass index was higher than those with a poor body mass index. It was determined that the nutritional awareness levels of adolescents with low body mass were also low, whereas the levels of nutrition awareness of adolescents with a normal body mass index were higher than those of students with an underweight body mass index. It has also been found that that obese individuals have low nutritional awareness when compared to individuals with a normal body mass (50). Therefore, it can be argued that individuals who do not have a healthy body mass have a lower awareness of healthy eating.

Another remarkable result regarding the correlations examined in the study is that parental neglect was significantly and negatively associated with obesity awareness in general. In other words, it was seen that parents who have attitudes and behaviors that can be defined as neglect have low awareness of their children’s obesity, nutrition and physical activity level. There are various findings in the literature that support these findings. In the cross-sectional study of Yin et al. (51), which included 844 parents, it was found that parents with low health literacy and healthy eating awareness have more obesogenic infant care behaviors, have limited or inadequate infant interaction during feeding time and engage in more controlling/pressuring feeding behaviors. In another study involving 3,164 children, it was determined that the children of parents who do not encourage their children to be physically active have a higher body mass index (52). In various studies, it has also been determined that the children of parents with a lack of awareness and inaccurate perceptions about healthy eating habits and high levels of inactivity have a high body mass index (53–55). Based on these findings, it is thought that parental neglect may cause a lack of knowledge and awareness about the child’s weight, nutrition and physical activity, which may make parental neglect a potential risk factor for obesity.

In the current study, in terms of the prediction of children’s body mass index, it was seen that in both underweight and overweight groups, parents’ perceptions about the weight of their children predicted the body mass index of the children. In the obese group, gender, eating speed, a history of obesity in the family, and parental perception of the child’s weight predicted the children’s body mass index in addition to neglectful behaviors of the parents. In the regression analysis, parents’ perceiving their children’s weight as normal, normal eating speed of the child and non-existence of a history of obesity in the family were taken as the reference groups. In other words, it is seen that parents in the underweight group accurately perceive their child as being underweight, and in the overweight and obese group, the parents accurately perceive their child’s weigh as overweight. On the other hand, in the overweight group, it is seen that parents consider the weight of their children as underweight, and this inaccurate perception of the parents is a predictor of the overweight body mass index in children. Thus, it can be suggested that parents of overweight children may have an inaccurate perception of their child’s weight and may underestimate their body mass. Parallel with this argument, in a meta-analysis study conducted by Rietmeijer-Mentink et al. (56), which included 51 studies and data on 35,103 children, it was shown that that more than 62% of overweight children were perceived as having normal weight by their parents.

A study has demonstrated that there may be a discrepancy between parents’ perceptions of their children’s weight and the actual situation. In particular, families of children who are obese may misperceive their children’s weight (57). Furthermore, it is postulated that parents’ misperceptions about their children’s weight status may have a detrimental effect on the formation of children’s healthy eating and physical activity habits (58). Skelton et al. (59) concluded that parents’ perceptions of their children’s weight status have a significant impact on children’s healthy weight control and lifestyle changes. Furthermore, it has been demonstrated that parent education programmes can positively influence parents’ nutritional habits, eating behaviors and nutritional approaches towards children (60). As in the present study, in a number of other studies, it was found that parents’ inaccurate estimation of their child’s body mass is more common if the child is overweight rather than obese (61, 62). On the other hand, in the study of Tschamler et al. (63), it was found that parents of obese and overweight children who underestimated their children’s weight were unconcerned about their weight. The reason why parents are not sufficiently concerned about the weight of their children’s may be due to the fact that they perceive their children’s weight to be normal.

In the present study, interestingly, neglectful behaviors of the parents was only found to be a significant predictor in the obese group. In other words, the level of neglect was higher in the parents of obese children. However, the parents of children in this group did not underestimate their child’s weight. Although they are aware of the excessive weight of their children, these parents do not have obesity awareness. Therefore, it can be argued that these parents are aware of their child’s weight status, but do not care enough about it or do not consider it as a health risk, and even if they do, they are unconcerned. An examination of the literature revealed that there are mixed results regarding the relationship between parental neglect and body mass in children. In a study by Bennet et al. (64), it was found that neglect predicted lower body mass in children, while obesity was not related to parental neglect. In contrast, there are also studies showing that parental neglect is associated with obese body mass in childhood (18, 65, 66). All these findings support the view that childhood obesity may also result from inadequacy or failure in the child’s care. However, directly linking obesity with neglect and suggesting that parents are responsible for obesity in their children may not be a holistic and accurate assessment. This is because, although the nutrition culture starts in the family, it is affected by many cultural, social and genetic factors. For instance, in the study, the presence of obesity in the family was found to be one of the factors that predicted obesity in children. This finding is also in line with the meta-analysis findings in the literature (67, 68). On the other hand, in a study involving 3,963 children and parents, Savaşhan et al. (69) found that 64.9% of parents of obese children were pleased with their child’s weight. Although it is less common today, the view that “strong and overweight” children are healthier in Turkish culture still reflects a dominant parenting approach. When the results obtained in the study are evaluated, it is thought that one of the reasons why parents are aware of their children’s weight, but do have not obesity awareness and do not perceive the weight of their children as a health problem, may be cultural.

In the present study, age and gender variables were also found to predict body mass in the obese group. In other words, being male and younger age predicted obesity. According to the Turkey Childhood Obesity Survey, obesity is more common in boys, while the prevalence of obesity decreases as age increases in school-aged children in Turkey according to the study of Kalkim et al. (70). It is thought that this finding related to age and gender may be related to gender-related social ideas about boys, and that these ideals may affect the feeding practices of parents. In addition, as age increases, concerns about weight increase in girls, and the desire to lose weight is more common than boys (71). Relationships between age, gender and body mass may also be developmental as a result of physiological factors such as hormones and body composition, as well as psycho-social factors. Along with age and gender, another factor that predicted obesity in the study was eating fast. This finding is in line with the findings in the literature on the subject (72–74). Eating fast is a risk factor for obesity, because when food is eaten fast, the nutrient and calorie intake is higher. Eating fast also makes it difficult to control weight. Therefore, slow eating not only reduces the need for food, but also creates a controllable eating behavior and constitutes a starting point on the way to healthy eating. In fast eaters, insulin secretion is less in the first minutes and at the end of the meal, which increases the risk of obesity (75).

## 5 Conclusion, limitations and future directions

When evaluated in general, the findings of this study suggest that parents’ perceptions of their children’s weight and their attitudes that can be defined as neglect may be related to their children having a body mass index categorized as being obese. While the association of neglect and obesity is a subject discussed in different contexts in the international literature, parental neglect in general has not been adequately addressed in the Turkish population; in other words, neglect remains a neglected issue in general, and its relationship with nutrition has not been examined. Therefore, it is thought that this study provides findings that can help provide a deeper perspective for understanding the risk factors of childhood obesity in Turkish society. The measures implemented in Turkey to prevent obesity are largely consistent with those employed globally. However, the measures are subject to periodic review in order to take into account the specific local and cultural needs. In both Turkey and the wider world, there is a strong focus on educational programmes, particularly for school-age children, health services to combat obesity, the promotion of physical activity, and the implementation of nutrition plans and policies. For instance, the “Türkiye Healthy Nutrition and Active Life Program” has been implemented with the objective of preventing obesity in Turkey. Similarly, in the United States, the “Let us Move!” initiative and programs such as “Change4Life” are implemented in the UK. The error has been rectified in the relevant section of the article. In this context, it is necessary to monitor the prevalence of overweight and obesity in children and their associated risk factors, to develop and implement action plans regarding obesity in children, to limit the marketing and advertising of unhealthy foods and beverages to children, and to implement the necessary prevention programs and strategies to promote healthier environments in schools (76).

However, there are some important issues that should be taken into consideration when evaluating the findings of the study. It is not an accurate approach to argue that obesity in children is directly caused by parents. Many genetic and biological factors are effective on obesity. An important point to be evaluated in obesity is the blood parameters of individuals. One of the limitations of this study is that other obesity-related factors and blood parameters were not included in this study. Broader assessments of mediators and moderators are needed to determine the environmental contexts in which neglect is associated with children’s weight. In order to examine the correlation we found between obesity and parental neglect in terms of causality, the issue needs to be examined especially in larger clinical samples. Parents’ lack of awareness about obesity, neglecting the child’s healthy diet and ignoring the possible risks for the future health of the child are argued to be among the most prominent factors that cause the failure of obesity interventions. As a result of the study, parental neglect was found to be associated with obesity according to the scale used, but obesity awareness is very important here. Parents may exhibit neglectful behavior, perhaps unknowingly, so nutrition education should be given more importance. It is evident that special consideration should be given to the family context and parental attitudes in obesity interventions. It is recommended that nutrition-related courses and activities be increased in schools. Furthermore, it is proposed that nutrition education be planned for both families and children. Finally, it is suggested that psycho-social studies be carried out. It is recommended that interventions for families be implemented in conjunction with medical interventions and should also include healthy nutrition and physical activity. In addition to their clinical implications, the findings also have important implications for educational settings. The phenomenon of neglect can be observed primarily in the school environment. Consequently, educators, school psychologists, and other practitioners working in schools, such as nurses, dietitians, and child development specialists, should consider weight problems in children as an indicator of parental neglect and should conduct intervention studies to enhance parental supervision of children at risk. It is recommended that the number of programmes and training initiatives designed to encourage healthy nutrition and physical activity be increased in schools. It is also important to raise awareness among parents in society about this issue. It is of paramount importance to provide psychological support to parents so that they may set a healthy example for their children.

## Data availability statement

The raw data supporting the conclusions of this article will be made available by the authors, without undue reservation.

## Ethics statement

The studies involving humans were approved by Ankara University Rectorate Ethics Committee. The studies were conducted in accordance with the local legislation and institutional requirements. Written informed consent for participation in this study was provided by the participants’ legal guardians/next of kin.

## Author contributions

TO: Software, Methodology, Conceptualization, Writing – review & editing, Writing – original draft. UB: Formal analysis, Data curation, Writing – review & editing, Writing – original draft. AU: Software, Methodology, Conceptualization, Writing – review & editing, Writing – original draft. AB: Writing – review & editing, Writing – original draft.
